# Identification of a novel site of interaction between ataxin-3 and the amyloid aggregation inhibitor polyglutamine binding peptide 1

**DOI:** 10.1177/1469066717729298

**Published:** 2017-08-29

**Authors:** Patrick D Knight, Theodoros K Karamanos, Sheena E Radford, Alison E Ashcroft

**Affiliations:** Faculty of Biological Sciences, University of Leeds, Leeds, UK

**Keywords:** Electrospray ionisation, ion mobility spectrometry-mass spectrometry, ataxin-3, polyglutamine, inhibition of amyloid assembly, polyQ binding protein 1, native mass spectrometry

## Abstract

Amyloid diseases represent a growing social and economic burden in the developed world. Understanding the assembly pathway and the inhibition of amyloid formation is key to developing therapies to treat these diseases. The neurodegenerative condition Machado–Joseph disease is characterised by the self-aggregation of the protein ataxin-3. Ataxin-3 consists of a globular N-terminal Josephin domain, which can aggregate into curvilinear protofibrils, and an unstructured, dynamically disordered C-terminal domain containing three ubiquitin interacting motifs separated by a polyglutamine stretch. Upon expansion of the polyglutamine region above 50 residues, ataxin-3 undergoes a second stage of aggregation in which long, straight amyloid fibrils form. A peptide inhibitor of polyglutamine aggregation, known as polyQ binding peptide 1, has been shown previously to prevent the maturation of ataxin-3 fibrils. However, the mechanism of this inhibition remains unclear. Using nanoelectrospray ionisation-mass spectrometry, we demonstrate that polyQ binding peptide 1 binds to monomeric ataxin-3. By investigating the ability of polyQ binding peptide 1 to bind to truncated ataxin-3 constructs lacking one or more domains, we localise the site of this interaction to a 39-residue sequence immediately C-terminal to the Josephin domain. The results suggest a new mechanism for the inhibition of polyglutamine aggregation by polyQ binding peptide 1 in which binding to a region outside of the polyglutamine tract can prevent fibril formation, highlighting the importance of polyglutamine flanking regions in controlling aggregation and disease.

## Introduction

Polyglutamine (polyQ) expansion diseases comprise a group of nine inherited protein aggregation disorders characterised by the deposition of proteins containing aberrantly expanded polyQ domains into amyloid fibrils.^1^ These disorders are characterised by a polyQ-length dependence on the age of onset of diseases, in which the age of disease onset correlates inversely with polyQ length.^1^ A different protein is involved in each disease within this class and the length of the polyQ domain required for fibril formation in vitro and disease onset varies, indicating that the sequence and context of the polyQ domain play an important role in determining aggregation propensity. Consistent with this, there is no sequence homology between the different proteins involved in polyQ aggregation disorders outside of their polyQ domains,^1^ and numerous biochemical and biophysical studies in vitro have shown the importance of the sequences flanking the polyQ tract in determining its aggregation propensity.^2–7^

**Figure 1.**
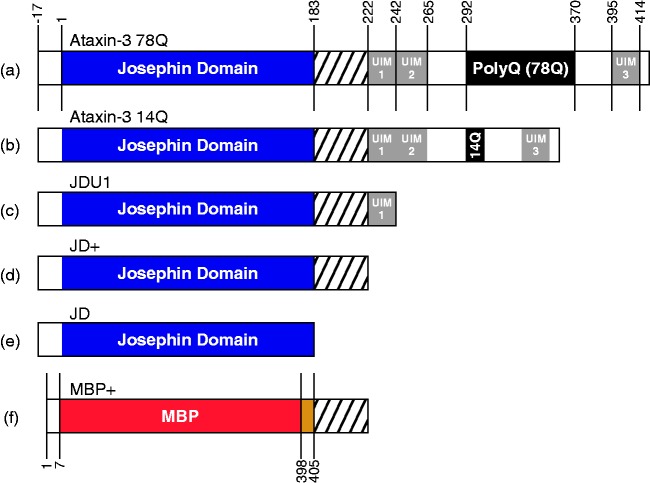
The architecture of ataxin-3. Ataxin-3 constructs used in these studies consist of a globular N-terminal domain (the JD, residues 1–182) and a largely disordered C-terminal tail containing three UIMs plus a polyQ domain. The constructs used are (a) ataxin-3 78Q; (b) ataxin-3 14Q; (c) JDU1, the JD plus the first UIM (residues 1–241); (d) JD+, the JD plus residues 183–221, (e) the JD alone (residues 1–182) and (f) MBP+, maltose binding protein linked to residues 183–221 of ataxin-3, separated by a TEV cleavage site. All ataxin-3 constructs (a–d) contain an N-terminal hexahistidine tag followed by a TEV cleavage site (LENLYFQG). JD: Josephin domain; UIMs: ubiquitin interacting motifs; polyQ: polyglutamine; MBP: maltose binding protein; TEV: Tobacco Etch Virus protease.

Machado–Joseph disease (MJD) is a member of the family of polyQ expansion diseases.^8^ In this disorder, disease occurs with the expansion of the polyQ domain beyond a threshold of 50 glutamine residues.^8,9^ Ataxin-3 is comprised of an N-terminal 21 kDa globular Josephin domain (JD) followed by two ubiquitin interacting motifs (UIM), a polyQ domain and, depending on the isoform, a third UIM.^10^ Each of these domains is linked by disordered regions of varying length (Figure 1(a)).

**Figure 2.**
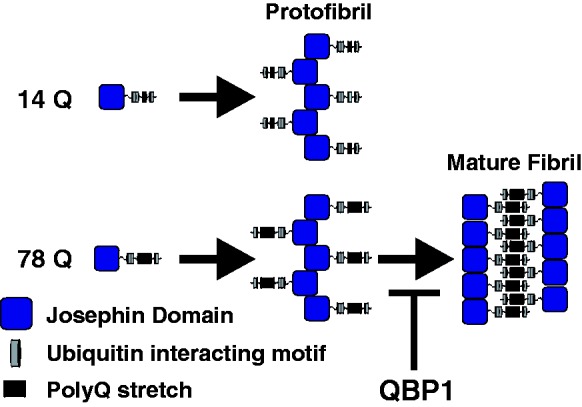
Ataxin-3 aggregation mechanism. Ataxin-3 aggregation proceeds by a two-step process: all ataxin-3 constructs undergo a Josephin domain-mediated initial aggregation step to produce protofibrils. However, only protofibrils derived from ataxin-3 with >55Q residues in the polyQ domain undergo the second step, a rearrangement which produces long, straight amyloid fibrils. The presence of the peptide QBP1 does not affect the first stage of aggregation, but inhibits the polyQ-dependent step.^11^ polyQ: polyglutamine; QBP1: polyQ binding peptide 1.

In vitro, the JD in isolation can undergo aggregation to form thioflavin-T (ThT) positive amyloid protofibrils.^11,12^ However, the presence of an expanded polyQ domain of >∼50 residues is required for maturation of the protofibrils into long straight fibrils typical of amyloid in a subsequent polyQ-dependent stage (Figure 2).^11^ Although an NMR structure has been solved for the N-terminal JD,^13^ little is known about the structure of the primarily disordered C-terminal ‘tail’ apart from the UIMs, which comprise two short α-helices.^14^ Despite detailed kinetic analyses of the aggregation of ataxin-3,^11,15–18^ why and how this protein undergoes two-phase aggregation remain unclear.

Screening of libraries for peptides able to bind to polyQ fusion proteins resulted in the discovery of an 11-residue sequence named polyQ binding peptide 1 (QBP1).^19^ QBP1 has been shown to be an effective inhibitor of polyQ aggregation both in vitro^19–22^ and in vivo.^23–25^ Surface plasmon resonance studies comparing the binding of QBP1 and Congo Red to a range of thioredoxin polyQ fusion proteins suggested that whereas Congo Red exhibits non-specific binding independent of the polyQ stretch, QBP1 binds specifically to the polyQ domain.^26^ However, as the thioredoxin-polyQ fusion protein is a non-disease protein, it has been suggested that this finding may not be significant to polyQ pathology.^27^ Other studies have also suggested that QBP1 interacts with polyQ protein monomers and prevents their pre-oligomerisation β-sheet conformational switch.^28^ Previous work has demonstrated that QBP1 inhibits the polyQ-dependent stage of ataxin-3 aggregation, while having no effect on polyQ-independent aggregation (Figure 2), but how the two species interact remained unknown.^11^ There is evidence for the efficacy of QBP1 as a therapeutic against amyloid disease in animal models;^23–25^ however, the lack of understanding of the mechanism of inhibition is one hurdle preventing the application of QBP1 or similar peptides as possible leads for the development of much-needed agents able to combat polyQ-related diseases.

Here we use nanoelectrospray ionisation-(ion mobility spectrometry)-mass spectrometry (ESI-(IMS)-MS) to examine the interaction of QBP1 with a series of ataxin-3 constructs that contain, or lack, different domains in order to identify the binding site(s) of the peptide for this polyQ-containing protein (Figure 1). The results demonstrate that QBP1 binds to monomers of ataxin-3 (78Q and 14Q) in a stoichiometric ratio. Surprisingly, similar binding was observed to truncated ataxin-3 variants lacking a polyQ domain, indicating that suppression of the polyQ-dependent stage of aggregation does not result from direct binding to the polyQ tract. Using truncation variants of ataxin-3, we identify a novel binding site for QBP1 as a 39-residue sequence immediately C-terminal to the JD. The results add insight into the nature of the conformational changes required for fibril formation of ataxin-3 and reinforce the findings in other systems that the aggregation of polyQ domains is highly dependent upon their flanking regions.^2–7^

## Experimental details

### Protein and peptide preparation

Ataxin-3 containing different polyQ lengths (ataxin-3 78Q and ataxin-3 14Q) and the truncated JD constructs (Figure 1) were expressed and purified as described previously.^12^ JDU1 and JD+ were created by placing a stop codon in the gene encoding ataxin-3 14Q using Q5 Quikchange mutagenesis (New England Biolabs Ltd., Herts., UK) and the primers shown in Supplementary Table 1. Sequences were inserted into the gene encoding ataxin-3 78Q within pET11a plasmids and proteins expressed in BL21(DE3)pLysS cells at 25℃ by auto induction.^12^ Protein expression was allowed to continue for 22 h, after which time bacteria were harvested. The ataxin-3 constructs were then purified using nickel affinity chromatography followed by size exclusion chromatography (S200 column) to create pure monomeric proteins. The resulting constructs are shown in schematic form in Figure 1.

The maltose binding protein (MBP) fusion protein was generated by appending the sequence for ataxin-3 residues 183–221 to the C-terminus of MBP with a TEV protease cleavable linker. Dr David Brockwell (University of Leeds) kindly provided a modified pMAL-c5X (New England Biolabs Ltd., Herts., UK) plasmid containing the BamA POTRA domains with the addition of an N-terminal 6 x His-tag (HT) and replacement of the thrombin cleavage site with a TEV cleavage site. Residues 183–221 of ataxin-3 were excised from the gene encoding ataxin-3 14Q using polymerase chain reaction (PCR), and then inserted in place of the BamA POTRA domains. The resulting construct was named MBP-183–221.

The UIM peptides 222–241 and 222–264 were purchased from Bachem AG (Bubendorf, Switzerland). N-terminally acetylated QBP1 (AcSNWKWWPGIFD) was purchased from Bio-Synthesis Inc. (Lewisville, TX, USA).

### PolyQ aggregation assays

Aggregation of the different ataxin-3 constructs was assayed by monitoring the fluorescence of ThT. Samples (10 μM protein) in the presence or absence of 50 μM QBP1 were mixed with 20 μM ThT in 250 mM ammonium hydrogen carbonate, 1 mM dithiothreitol, pH 7.8. In all, 100 µL of sample was added to a single well of a 96-well plate (catalogue number 3881, Corning, Sigma-Aldrich, Dorset, UK). Fluorescence was monitored using a FluoStar Optima or Omega plate reader (BMG Labtech, Aylesbury, Bucks, UK). Excitation was at 440 nm and emission was read at 475 nm. The plate was held at 37℃ without shaking for the duration of the experiments.

Negative stain transmission electron micrographs (EMs) of the end point of the fibrillation reaction (51 h) were acquired using a JEM 1400 transmission electron micrograph (JEOL Ltd., Tokyo, Japan). Samples were pipetted onto Formvar-carbon grids and negatively stained with 4% (w/v) uranyl acetate.

### ^1^H-nuclear magnetic resonance

T1ρ experiments were performed on samples containing 50 μM QBP1 in 250 mM ammonium hydrogen carbonate, 10% (v/v) D_2_O and 10 μM protein. Data were acquired on a 600 MHz NMR magnet (Oxford Instruments, Abingdon, UK) using a QCI-P-cryoprobe and an Avance III HD console (Bruker Corpn., Coventry, UK) using a 100 ms spin-lock. The data were acquired and processed with Bruker TopSpin, NMRPipe and CcpNMR analysis software.

### Nano-ESI-(IMS)-MS

ESI-(IMS)-MS experiments were performed on a Synapt HDMS Q-ToF instrument equipped with travelling wave IMS situated between the two analysers (Waters UK Ltd., Wilmslow, Cheshire, UK). Samples were prepared as 10 μM protein and 50 μM QBP1 in 250 mM ammonium hydrogen carbonate, 1 mM dithiothreitol, pH 7.8. The samples were infused via gold-palladium coated borosilicate nano-capillaries fabricated in-house. Typical instrument settings were: capillary voltage 1.5–1.7 kV, sampling cone 60 V, trap collision energy 15 V and transfer collision energy 25 V. Calibration of the m/z range was achieved on NaI clusters (2 mg/mL 1:1 v/v propan-2-ol:water NaI). Data were acquired, processed and visualised using the software provided with the instruments (MassLynx 4.1 and Driftscope 2.5).

Ion mobility data were acquired simultaneously with MS measurements. Travelling wave IMS calibration was performed using a range of standards from the published list of protein calibrants.^29^ The specific calibrants used here were cyctochrome C, concanavalin A, β-lactoglobulin, alcohol dehydrogenase and avidin. Each protein calibrant was analysed three times and the average collision cross-section (CCS) calculated.

## Results

### Ataxin-3 aggregation inhibition by QBP1 is maintained in volatile solvents

**Figure 3.**
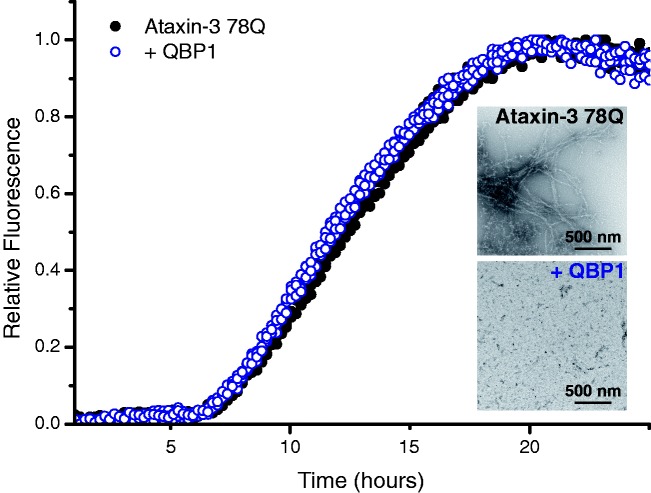
Self-assembly of ataxin-3 78Q monitored by ThT fluorescence. Protein aggregation was measured in the absence (black solid circles) or presence (blue open circles) of a five-fold molar excess of QBP1. After a lag-time of ∼5 h, protofibrils form which then convert into amyloid fibrils (inset top: TEM image). In the presence of the peptide inhibitor QBP1, the ataxin-3 protofibrils form (blue open circles) but do not convert into full-length amyloid fibrils (inset lower: TEM image). Both TEM images were taken at t = 51 h. ThT: thioflavin-T; QBP1: polyQ binding peptide 1; TEM: transmission electron micrograph.

The progress of aggregation of ataxin-3 78Q in the presence or absence of a five-fold molar excess of QBP1 in the ESI-MS compatible buffer, 250 mM ammonium hydrogen carbonate, pH 7.8 is shown in Figure 3. Consistent with previous results,^11,12^ aggregation proceeds via a ThT-sensitive phase, which results in the formation of protofibrils, followed by a ThT-insensitive second phase in which the final long, straight amyloid fibrils form (Figure 3, inset). As expected,^11^ addition of QBP1 to ataxin-3 78Q had no effect on the formation of protofibrils, as measured by the ThT fluorescence assay, but prevented conversion to mature fibrils (Figure 3). Thus, the aggregation processes of ataxin-3, and the inhibition of these processes by QBP1, are maintained under MS-amenable conditions.

### ESI-MS reveals a novel binding site for QBP1 and ataxin-3 independent of the polyQ tract

**Figure 4.**
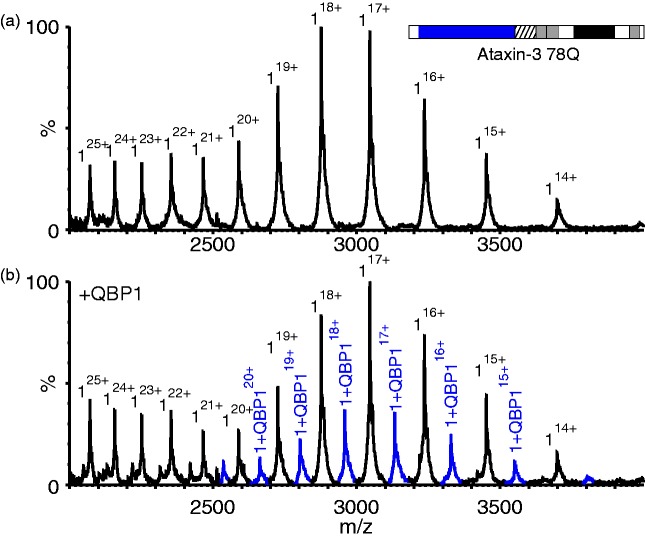
QBP1 binds to compact conformers of ataxin-3 78Q. ESI mass spectra of (a) ataxin-3 78Q (51,751.0 Da; black peaks) and (b) ataxin-3 78Q in the presence of a five-fold molar excess of QBP1 (mass 1477.6 Da). The peaks corresponding to a 1:1 ataxin-3 78Q: QBP1 complex (53,228.6 Da) are shown in blue. Masses of all proteins and their complexes are shown in Supplementary Table 2. QBP1: polyQ binding peptide 1; ESI: electrospray ionisation.

To determine how QBP1 prevents amyloid fibril formation of ataxin-3 78Q, the ability of different variants, lacking one or more domains, to bind QBP1 was analysed using ESI-MS. The native ESI mass spectrum of ataxin-3 78Q is shown in Figure 4(a). The spectrum reveals that under the conditions employed ataxin-3 78Q is primarily monomeric (all theoretical and observed masses are detailed in Supplementary Table 2), and results in two charge state distributions arising from compact ((M + 14H)^14+^ to (M + 20H)^20+^) and more expanded (≥(M+21H)^21+^) conformations, consistent with previous results (Figure 4(a)).^12,30^ Such profiles tend to indicate the protein’s conformational characteristics in solution.^31^ The addition of QBP1 to ataxin-3 78Q leads to the formation of a 1:1 complex between the peptide and protein (Supplementary Table 2) (Figure 4(b)). No further binding was observed when the concentration of QBP1 was raised to a 16-fold molar excess suggestive of specific binding (data not shown). Additionally, QBP1 was found to bind only to the more compact conformers of ataxin-3 78Q (i.e. to the 14^+^ to 20^+^ charge state ions), while no binding was observed for the more highly charged ions (≥21^+^).

**Figure 5.**
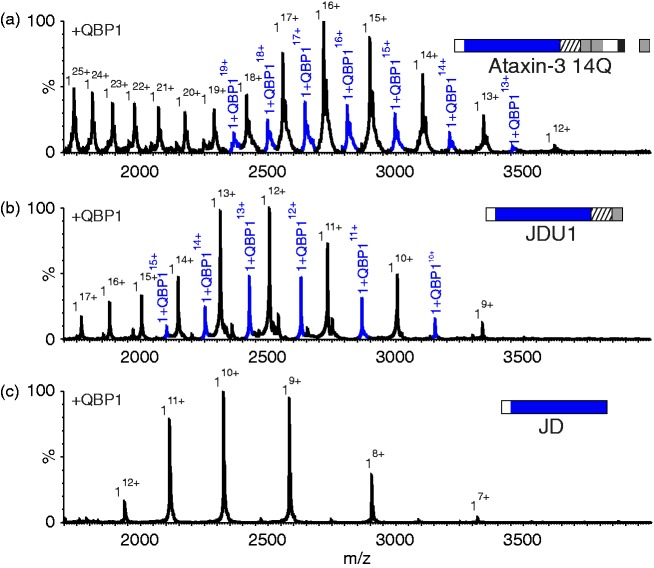
QBP1 interacts with ataxin-3 constructs lacking a polyQ domain, but not with the JD alone. ESI mass spectra of (a) ataxin-3 14Q (mass 43,451.3 Da; mass of 1:1 complex 14Q:QBP1, 44,929.1 Da); (b) ataxin-3 residues 1–241 (JDU1) (mass 30,046.7 Da; mass 1:1 complex JDU1:QBP1, 31,522.5 Da); and (c) the JD alone (ataxin-3 residues 1–182, mass 23,237.2 Da), each in the presence of a five-fold molar excess of QBP1. Peaks corresponding to 1:1 complexes of protein: QBP1 are shown in blue. Masses of all proteins and their complexes are shown in Supplementary Table 2. QBP1: polyQ binding peptide 1; polyQ: polyglutamine; JD: Josephin domain.

Given that QBP1 was selected against a 62-residue polyQ sequence^19^ and has been shown not to bind polyQ segments of 19 residues in length,^26^ the ability of QBP1 to bind ataxin-3 of non-pathological length (14Q; Figure 1(b)) was tested next using ESI-MS. The results (Figure 5(a)) showed that ataxin-3 14Q is also able to bind to QBP1, with a 1:1 binding stoichiometry (Supplementary Table 2) being observed both when a five-fold (Figure 5(a)) and 16-fold (Supplementary Figure 1) excess of peptide are added. Similar to its longer polyQ counterpart, only the more compact of the two protein conformer populations observed was found to bind to QBP1.

Further investigations using a truncation variant of ataxin-3 comprising the JD with UIM1 only (i.e. residues 1–241) (JDU1; Figure 1(c)), revealed that the deletion of all residues C-terminal to the UIM does not alter binding (Figure 5(b) and Supplementary Table 2). This construct does not contain a polyQ tract, revealing the surprising results that a polyQ domain is not required for QBP1 binding. Importantly, a complex between QBP1 and the JD alone (consisting of residues 1–182; Figure 1(e)) was not observed (Figure 5(c) and Supplementary Table 2), confirming the specificity of binding to JDU1 and the longer ataxin-3 constructs. Together, the results suggest that QBP1 binds to residues 183–241 of ataxin-3. Given that a 1:1 complex is observed for all of the constructs which bind QBP1, even when an excess of peptide is added, it is likely that the same binding site is mediating the interaction in all of the constructs.

**Figure 6.**
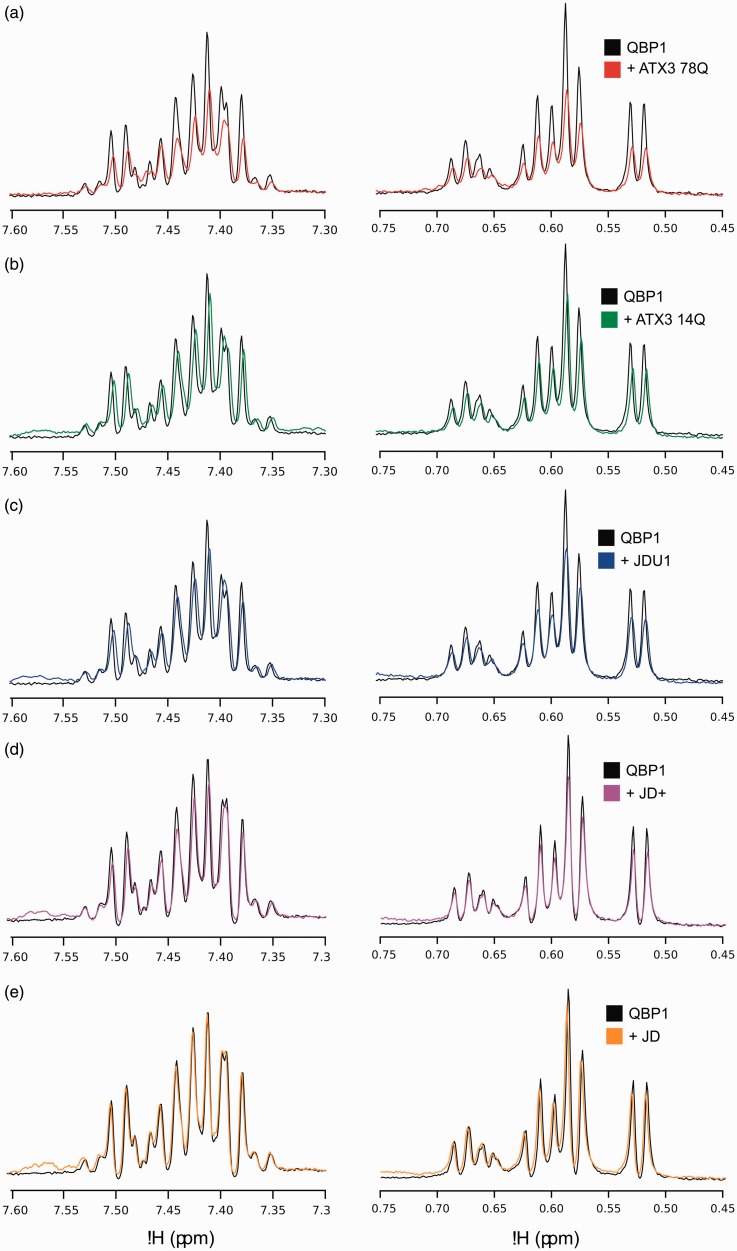
Solution-phase ^1^H-NMR confirms QBP1 binds to ataxin-3 78Q, ataxin-3 14Q, JDU1 and JD+, but not JD. T1ρ NMR spectra for QBP1 alone (black) and in the presence of (a) ataxin-3 78Q (red), (b) ataxin-3 14Q (green), (c) ataxin-3 residues 1–241 (JDU1; blue), (d) ataxin-3 residues 1–221 (JD+; purple) and (e) the Josephin domain alone residues 1–183 (JD; orange). A reduction in signal intensity (a–d) indicates an interaction between the peptide and the protein. NMR: nuclear magnetic resonance; QBP1: polyQ binding peptide 1.

To support the unexpected observation that QBP1 binds to a region of ataxin-3 distant to the polyQ region, T1ρ NMR experiments were performed to provide orthogonal, solution-phase information. In the T1ρ experiment, binding of a low molecular mass ligand to a high molecular mass partner results in line broadening (and therefore loss of signal intensity) due to the increased ‘tumbling time’ of the ligand in the complex in a manner that depends on the exchange rate and the size of the complex.^32^ The T1ρ technique is particularly sensitive for weak interactions where chemical shift perturbations upon binding are not observed due to the low population of the complex. After integration of the resonances arising from the methyl region of the T1ρ spectrum (0.45–0.75 ppm), the results of these experiments showed an attenuation of the peptide signals when QBP1 is bound to ataxin-3 78Q (24% loss of signal; Figure 6(a)), ataxin-3 14Q (22% signal loss; Figure 6(b)), JDU1 (14% signal loss; Figure 6(c)) and JD+ (8% signal loss; Figure 6(d)), but no loss in signal when QBP1 was mixed with JD alone (Figure 6(e)). The decreased effect of signal attenuation shown in Figure 6(a) to (d) is consistent with the decreasing molecular weight of these complexes in comparison to ataxin-3 78Q. These experiments thus show that QBP1 binds to the ataxin-3 constructs ataxin-3 78Q, ataxin-3 14Q, JDU1 and JD+ in solution, as well as retaining their bound state in the gas-phase.

### Residues 182–221 of ataxin-3 bind QBP1

The results presented above indicate that while the JD (residues 1–182) does not bind QBP1 (Figure 5(c)), extension of this construct by the addition of residues 183–241 (JDU1; Figure 1(c)) restores the ability of the protein to bind QBP1 (Figure 5(b)). The region 183–241 must thus confer binding. This region contains a 39-residue disordered sequence followed by the first UIM of ataxin-3 (Figure 1). Interestingly, previous studies of ataxin-3^33,34^ have suggested that UIMs can be involved in the aggregation process and may inhibit aggregation of polyQ proteins. Binding of QBP1 to this region may thus prevent conformational changes required for conversion of protofibrils into fully assembled amyloid fibrils involving self-association of the polyQ region.

**Figure 7.**
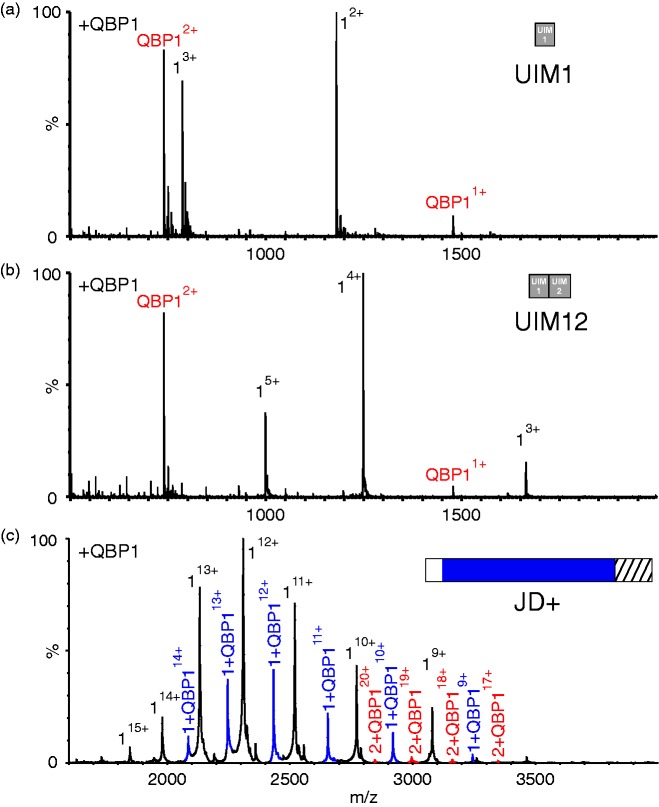
Ataxin-3 residues 183–221 are sufficient for QBP1 binding. ESI mass spectra of QBP1 (mass 1477.6 Da) with (a) a synthetic peptide equivalent to residues 222–241 (UIM1) of ataxin-3 (mass 2358.5 Da; mass of 1:1 complex UIM1:QBP1, 3836.1 Da; (b) a synthetic peptide equivalent to residues ataxin-3 residues 222–264 of ataxin-3 (UIM12; mass 4990.4 Da; mass of 1:1 complex UIM12:QBP1, 6468.0 Da). No evidence for protein:QBP1 binding is observed in (a) or (b). (c) ESI mass spectrum of ataxin-3 1–221 (JD+; mass 27,704.4 Da) in the presence of a five-fold molar excess of the peptide QBP1. Peaks corresponding to a 1:1 complex JD+:QBP1 (mass 29,182.2 Da) are shown in blue. Peaks corresponding to a 1:1 complex of protein dimer to QBP1 (mass 56,886.4 Da) are shown in red. Masses of all proteins and their complexes are given in Supplementary Table 2. QBP1: polyQ binding peptide 1; ESI: electrospray ionisation.

To explore whether the UIMs of ataxin-3 confer binding to QBP1, ESI-MS was carried out on peptides comprising the sequence of the first UIM (residues 222–241; UIM1) of ataxin-3, or the first and second UIMs (residues 222–264; UIM12), in the absence or presence of equimolar QBP1. These experiments (Figure 7(a) and (b)) showed no evidence for any interaction between these peptides and QBP1, despite the peptides adopting helical structure (revealed by far UV CD; Supplementary Figure 2 and Table 3) consistent with their native structure. Conversely, an ataxin-3 construct (residues 1–221) which contains the JD and the subsequent 39-residue disordered sequence (JD+, Figure 1(d)) was found to interact with QBP1, forming a 1:1 complex as was observed for the longer ataxin-3 constructs (Figure 7(c) and Supplementary Table 2).

**Figure 8.**
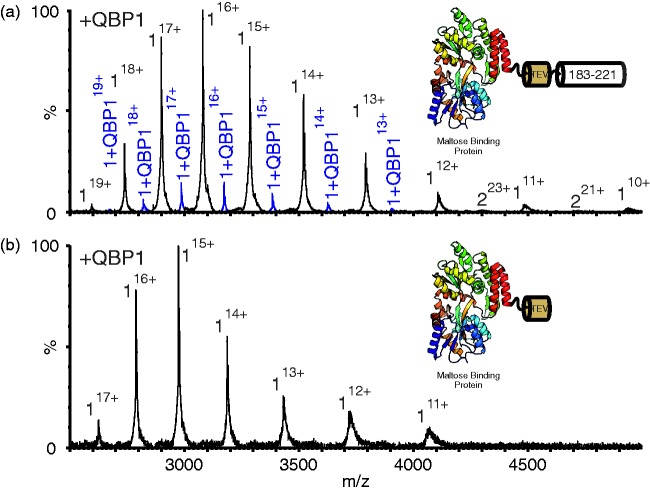
Residues 183–221 of ataxin-3 bind QBP1. (a) ESI mass spectrum of the construct of MBP-183–221 (mass 49,207.6 Da) in the presence of equimolar QBP1 (mass 1477.6 Da). Peaks corresponding to a 1:1 complex of protein:QBP1 (mass 50,685.2 Da) are shown in blue. Inset shows a schematic of MBP-183–221. This contains MBP (PDB 1LLS^35^), linked to residues 183–221 of ataxin-3 separated by a TEV cleavage site (see Methods). (b) ESI mass spectrum of the TEV-cleaved Maltose Binding Protein ataxin-3 183–221 fusion protein (mass 44,596.4 Da) in the presence of QBP1. No peaks corresponding to a protein:QBP1 complex are observed. Inset shows a schematic of MBP-183–221 following cleavage of residues 183–221. Masses of all proteins and their complexes are given in Supplementary Table 2. QBP1: polyQ binding peptide 1; ESI: electrospray ionisation; MBP: maltose binding protein.

The data from these experiments suggest that residues 183–221 are necessary and sufficient for interaction of ataxin-3 with QBP1. To examine whether residues 183–221 alone are sufficient for binding, independent of the JD, the sequence 183–221 of ataxin-3 was appended C-terminally to MBP separated by a TEV cleavage site (Figures 1(f) and 8, inset). MBP provides a stable solubilisation domain and has been used previously in the investigation of truncated ataxin-3 constructs.^36^ Remarkably, native ESI-MS of this protein (MBP-183–221) in the presence of QBP1 resulted in a 1:1 complex (Figure 8(a) and Supplementary Table 2). Treatment with TEV protease to remove the ataxin-3-derived residues abrogated the QBP1 interaction, indicative of a specific interaction between residues 183–221 of ataxin-3 and QBP1 (Figure 8(b)). These data demonstrate that residues 183–221 are both necessary and sufficient for interaction with QBP1 and that this binding competence is maintained even when the residues are removed from their native protein context.

### QBP1 binding does not alter ataxin-3 conformation

**Figure 9.**
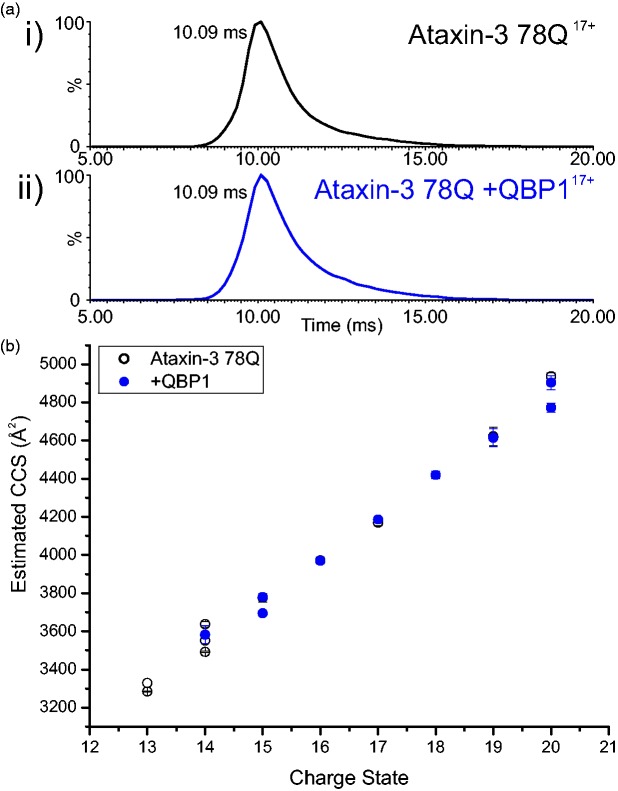
ESI-(IMS)-MS indicates that ataxin-3 78Q does not undergo any significant change in CCS upon binding QBP1. (a) ESI-(IMS)-MS arrival time distribution for the 17^+^ charge state ions of (i) ataxin-3 78Q alone, and (ii) ataxin-3 78Q:QBP1 1:1 complex; (b) CCS calculated from ESI-(IMS)-MS data for 13^+^ to 20^+^ charge state ions for ataxin-3 78Q alone (white circles) and the ataxin-3 78Q:QBP1 complex (blue circles). n.b. The CCS measurements for the 15^+^ and 16^+^ ions of ataxin-3 78Q and ataxin-3 78Q:QBP1 complex are indistinguishable. ESI-(IMS)-MS: electrospray ionisation-(ion mobility spectrometry)-mass spectrometry; CCS: collision cross-section; QBP1: polyQ binding protein 1.

Several inhibitors of amyloid aggregation have been shown to alter the conformation of the bound protein.^37^ To investigate whether this is the case for the QBP1:ataxin-3 complex observed here, ESI-(IMS)-MS was used to measure the CCS of ataxin-3 78Q in the absence or presence of QBP1. The CCS can be measured from the IMS-MS data based on the feature that ions of the same charge and m/z will be separated if their physical size differs, with the larger ions travelling more slowly through the gas-filled IMS cell.^29,38,39^ The results showed a very similar arrival time distribution for the 17^+^ ions for both ataxin-3 78Q and the ataxin-3 78Q:QBP1 complex (Figure 9(a)). Indeed, across all charge states the CCS values for bound and unbound protein are not significantly different (Figure 9(b)). Similar results were observed for all other ataxin-3 constructs able to bind QBP1 (Supplementary Figure 3). Thus, at least as measured by ESI-(IMS)-MS, no substantial alterations in the conformational properties of the disordered ‘tail’ of ataxin-3 occur when QBP1 binds to the region 183–221.

## Conclusions

Searching for inhibitors of protein aggregation is essential to elucidate in more detail how proteins self-assemble into amyloid fibrils, and to further our understanding of how protein aggregation causes disease. The peptide QBP1 is one such inhibitor, which was selected against polyQ-containing proteins^19^ and shown to inhibit the aggregation of polyQ fusion proteins and ataxin-3 in vitro and in vivo.^11,19–25,28^ Here we present the surprising finding that while QBP1 prevents amyloid formation of ataxin-3 78Q, it does so via a novel binding site that is distal to the polyQ tract. By creation of a series of ataxin-3 constructs in which each protein differs by one or more domains (Figure 1), and analysis of the ability of the resulting constructs to bind QBP1 using ESI-MS, we show that ataxin-3 residues 183–221 are both necessary and sufficient for binding to QBP1. This region has not previously been considered to have a role in QBP1 binding. Importantly, since only 1:1 binding is observed even in the presence of a 16-fold molar excess of peptide, the results presented suggest that while expanded polyQ can bind QBP1, such binding must be precluded in the ataxin-3 proteins studied here. This effect may be caused by conformational changes in which the region 183–221 occludes the polyQ binding site, although such a change is not detectable using IMS-MS, possibly because the overall size of the protein does not alter significantly, or because of structural changes in this intrinsically disordered region in the gas-phase.^40^ Nonetheless, the results presented support a model in which the region 183–221 plays a vital role in the progression of ataxin-3 aggregation from the JD-dependent initial phase of protofibril formation to the second stage in which polyQ-dependent amyloid fibrils form (Figure 2). The importance of residues distant from the polyQ domain in mediating aggregation reiterates existing evidence for the importance of flanking regions of polyQ-containing proteins upon their aggregation into amyloid. For example, it has been demonstrated that the JD plays an important role in polyQ-dependent aggregation of ataxin-3.^18,41^ For other polyQ proteins, the sequence context of a polyQ domain has also been shown to influence the threshold at which the protein becomes a pathogenic expansion.^1^

While residues 183–221 of ataxin-3 have not been considered previously as possible binding sites for QBP1, there is literature precedent for the involvement of this region in ataxin-3 aggregation. The so-called ‘central flexible region’ of ataxin-3, consisting of residues 183–291, has been shown (using ThT assays) to increase the rate of aggregation of ataxin-3, while the addition of these residues as a soluble protein reduces the rate of aggregation through disruption of the aggregation process via competition.^33,34^ Together with the results presented here, a model for QBP1 inhibition of amyloid formation of ataxin-3 emerges in which binding to the region 183–221 prevents an interaction between the C-terminal flexible domain and other regions of ataxin-3 that is essential for the second polyQ-dependent stage of aggregation into amyloid. These interactions could be mediated by the UIMs, as suggested by Papaleo and co-workers.^33^ While direct binding of QBP1 to peptides equivalent to the UIMs of ataxin-3 could not be detected using ESI-MS, it cannot be ruled out that the effect of QBP1 on aggregation of full length ataxin-3 may be mediated in part by the UIMs, which are immediately adjacent to the newly identified binding site.

When subjected to a blast search (BlastP) against the human proteome (SwissProt) ataxin-3 residues 183–221 show no sequence homology to proteins other than ataxin-3, suggestive of a unique ability of residues 183–221 of ataxin-3 to bind QBP1. Importantly, aligning 183–221 to the glutathione-S-transferase (GST)-Q62 construct, against which QBP1 was originally raised,^19^ also generated no hits, discounting that residues 183–221 share any homology to the GST tag used for its selection. Residues 183–221 are not predicted to have a high aggregation propensity as determined by TANGO,^42–44^ by previous research on the central flexible region of ataxin-3 (residues 182–291),^33,34^ and by other amyloid prediction algorithms.^34^

While no conformational change in ataxin-3 78Q was observed upon the binding of QBP1, the MS approach employed here is limited to the observation of monomeric and early-stage oligomers in the polyQ independent aggregation pathway. The β-sheet transition previously hypothesised to be inhibited by QBP1^28^ is likely to occur in higher order oligomers or protofibrils that were not observed in this investigation.^11^ It is conceivable that QBP1 might prevent ataxin-3 from transitioning into these states at later time points through the interaction described here.

The importance of residues outside the polyQ domain in mediating the inhibition of aggregation reiterates the growing body of evidence for the importance of flanking domains in modulating and controlling amyloid aggregation. The results presented here thus have importance in terms of future design of small molecules or peptidomimetics to combat polyQ diseases. In the quest for potential aggregation inhibitors, binding sites other than the polyQ region will require further evaluation, including identifying and targeting flanking domains that are involved in aggregation. A further goal to pursue is determining the residues responsible for binding QBP1 to ataxin-3 constructs. This would require the design and synthesis of a range of peptides in order to pin-point the origin and extent of specificity. Despite the fact that there is a long way to go before satisfactory treatments are developed for polyQ diseases, the results presented here highlight the importance of residues 183–221 in ataxin-3 aggregation, and highlight the power of MS and MS-based approaches to identify ligand binding sites when combined with a series of rationally designed truncation variants. Moreover, the results identify a critical role of residues 183–221 in ataxin-3 aggregation and raise a number of fascinating questions about how and why this sequence of the protein is critical for the conversion of protofibrils formed by self-association of the JD to amyloid fibrils into the second polyQ-dependent step of ataxin-3 aggregation.

## Supplementary Material

Supplementary material
