# Supplementary material for: Identification of a novel site of interaction between ataxin-3 and the amyloid aggregation inhibitor polyglutamine binding peptide 1
Source: Eur J Mass Spectrom (Chichester). 2017 Aug 29;24(1):129–40. doi: 10.1177/1469066717729298 (PMC6134688; doi:10.1177/1469066717729298)
Supplement: Supplementary material [file supplementary_material.pdf]

Identification of a novel site of interaction between ataxin-3 and the amyloid aggregation inhibitor polyglutamine binding protein 1

Patrick D. Knight, Theodoros K. Karamanos, Sheena E. Radford* and Alison E. Ashcroft*

The Astbury Centre for Structural Molecular Biology, School of Molecular and Cellular Biology, University of Leeds, Leeds, United Kingdom, LS2 9JT, UK

*= corresponding authors: a.e.ashcroft@leeds.ac.uk; s.e.radford@leeds.ac.uk

Supplementary Information

Supplementary Table 1: Primers used in this investigation.

Construct	Primers used
JDU1	CGCCAAGAAATTGACTGATAAGATGAGGAAGCAGATCTCCGCAGGG<br>CCCTGCGGAGATCTGCTTCCTCATCTTATCAGTCAATTTCTTGGCG
JD+	TAATTAGACGAAGATGAGCAG<br>CATTCCTGAGCCATCATTTG
MBP+	ATTACTTGCGGATCCGTCCAACAGATGCATCGACCAAAAC<br>AGGACTAGAGAATTCCTTACATTCCTGAGCCATCATTTGCTTC

Expression and purification of MBP-183-221

MBP-183-221 plasmid was transformed into BL21[DE3] cell. A single colony was sued to inoculate LB starter cultures containing 100 µg/ml carbenicillin which were grown overnight (37 °C, 200 rpm). The starter cultures were used to inoculate 1L LB flasks containing 100 µg/ml carbenicillin. Cells were grown at 37 °C, 200 rpm until the OD600 reached 0.6 after which expression was induced by the addition of 1 mM IPTG. After 4 hours of expression the cells were harvested by centrifugation (5000g, 15 min). The subsequent purification followed the protocol for ataxin-3, as described in the main text.

Expression and purification of His-tagged TEV protease

Vector pMHTDelta238 containing His-tagged TEV fused with MBP which is removed *in vivo* by autocleavage,[1] was obtained from DNASU (Clone TvCD00084286). This vector was transformed into BL21-CodonPlus[DE3]-RIPL cells (Stratagene, UK). Cells were grown in LB medium containing 50 µg/mL kanamycin at 37 °C with shaking (200 rpm) until the culture reached an OD600 of ~0.6. The temperature was then lowered to 30 °C and expression induced with 0.5 mM IPTG. Following expression (~4 h) cells were harvested by centrifugation, re-suspended in 25 mM sodium phosphate buffer, pH 8.0, 200 mM NaCl, 10% (v/v) glycerol, 25 mM imidazole, 1 mM PMSF, 2 mM benzamidine, ~0.02 mg/ml DNase (Sigma, UK), and lysed by sonication (6 x 30 s bursts with 1 min cooling on ice between each sonication). Following centrifugation to remove cell debris (20 mins, 4 °C, 39000 g), the lysate was applied to Ni^{2+} Sepharose beads (GE Healthcare) and washed twice with 25 mM sodium phosphate buffer, pH 8.0, 200 mM NaCl, 10% (v/v) glycerol, 25 mM imidazole. His-tagged TEV was eluted with 25 mM sodium phosphate buffer, pH 8.0, 200 mM NaCl, 10% (v/v) glycerol, 500 mM imidazole. The eluate was filtered (0.2 µM syringe filter, Sartorius, UK) and gel filtered on a HiLoad Superdex 75 26/60 column (GE Healthcare) equilibrated with 25 mM sodium phosphate buffer, pH 8.0, 200 mM NaCl, 25 mM imidazole, 10% (v/v) glycerol, 5 mM β-mercaptoethanol. Peak fractions were concentrated to ~1 mg/mL using Vivaspin 20 (5 kDa MWCO) concentrators (Sartorius, UK), aliquoted, snap-frozen in liquid nitrogen and stored at 80 °C.

TEV cleavage of MBP-183-221

His-tagged TEV protease was added at a 1:10 TEV:MBP-183-221 molar ratio and incubated for 1 hour 45 minutes at 20 °C. The TEV concentration was 20 µM during the digestion and was subsequently diluted for analysis. These conditions were found to be optimal for the digestion, longer digest times and higher concentrations resulted in poor protein recovery.

Supplementary Table 2: Expected and observed masses for proteins and peptides used in this investigation. All values quoted are in Da and reflect the masses determined in the data shown in this paper. Expected masses are isotopically averaged masses.

**Construct**	**Expected mass**	**Observed mass**	**+QBP1 expected mass**	**+QBP1 observed mass**
**Ataxin-3 78Q**	51751.0	51754.1 ± 6.9 Da	53228.6	53231.0 ± 8.4 Da
**Ataxin-3 14Q**	43451.5	43457.3 ± 0.4 Da	44929.1	44935.9 ± 0.4 Da
**JDU1**	30044.9	30046.7 ± 2.9 Da	31522.5	31526.9 ± 0.2 Da
**JD+**	27704.4	27707.0 ± 0.6 Da	29182.0	29185.8 ± 0.3 Da
**JD**	23237.2	23239.4 ± 5.5 Da	24714.8	
**MBP+**	49207.6	49206.5 ± 0.6 Da	50685.2	50686.8 ± 0.3 Da
**MBP (post TEV)**	44596.4	44595.0 ± 13.9 Da	46072.6	
**UIM12**	4990.4	4991.2 ± 2.6 Da	6468.0	
**UIM1**	2358.5	2357.2 ± 0.1 Da	3836.1	
**QBP1**	1477.6	1478.2 ± 1.9 Da		

Supplementary Figure 1: QBP1 binds to ATX3 78Q in a 1:1 ratio, even at high excesses of peptide. The nESI-MS spectrum of ataxin-3 78Q in the presence of a 16-fold molar excess of QBP1 (160 µM QBP1, 10 µM ataxin-3 78Q) shows the same 1:1 protein:peptide complex as observed at lower QBP1 concentrations. No evidence is seen for any higher order complexes.

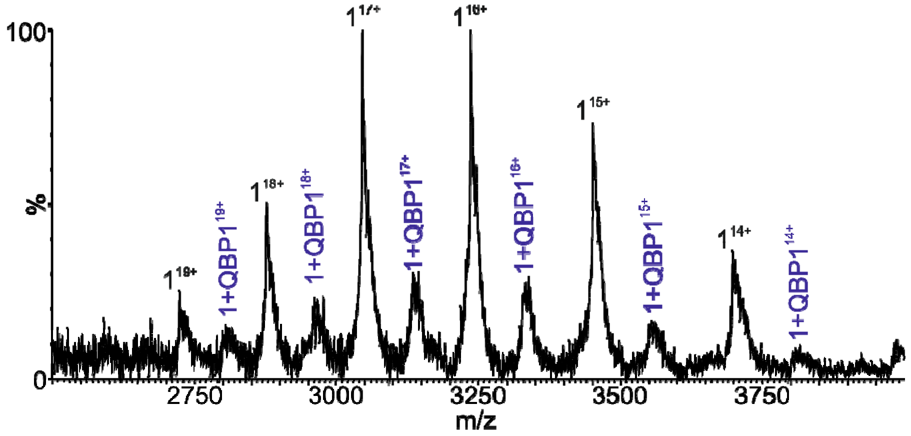

Supplementary Figure 2: Far UV CD shows that secondary structure is maintained in UIM peptides. The far UV CD spectra of UIM1 and UIM12 have negative maxima at 205 nm and 221 nm suggesting a mixture of α-helical and random coil structure. Spectra of UIM1 (solid line) and UIM12 (dashed line) were recorded at 20 °C.

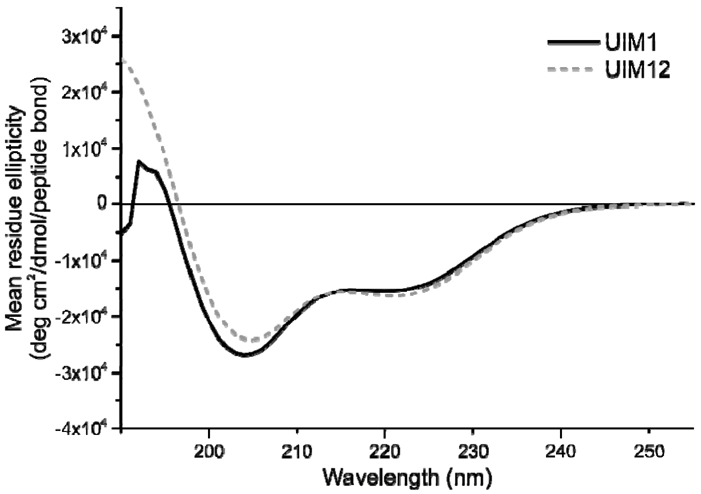

Supplementary Table 3: Helical secondary structure content of UIM1 and UIM12 determined from far UV CD data shown in Supplementary Figure 2. These data were analysed using the CDSSTR[2] method on Dichroweb[3]. The data are consistent with the NMR structures which suggest that UIM12 has 53% helical residues[4], while UIM1 is 65% helical[4].

	Experimental α-helical content	Measured α-helical content (*Song et al.*)
UIM1	**64%**	**65%**
UIM12	**59%**	**53%**

Far UV Circular Dichroism

The peptides UIM1 and UIM12 were each suspended in 50 mM sodium phosphate buffer, pH 8.0 at 0.25 mg/mL and 0.11 mg/mL, respectively. As the peptides lacked residues which absorb at 280 nm, the concentrations of the peptides were determined from the absorbance at 205 nm on a UV-1800 dual beam spectrophotometer (Shimadzu, Tokyo, Japan) following the Stokes method[5]. Far UV CD spectra were recorded over the range 180-260 nm at 20 °C with a Peltier temperature controller on a Chirascan spectrophotometer (Applied Photophysics, Leatherhead, Surrey, UK) with 4 nm bandwidth, in a 1.0 mm cuvette. Each spectrum was recorded three times and the average of the three scans is shown. The secondary structure content of each of the peptides was calculated from these data using the CDSSTR[2] method available on Dichroweb[3].

Supplementary Figure 3: The Arrival Time Distributions (ATDs) of ataxin-3 variants, either with a short polyQ domain or lacking a polyQ domain, are unaffected by QBP1 binding. The ATDs were recorded for all observed ions for each of the ataxin-3 constructs (ataxin-3 14Q, JDU1 and JD+) that showed binding to QBP1. The ATD for the most abundant ion in each case is illustrated as indicated in the figure. In each case the maximum for the ATDs for the bound and free ataxin-3 construct overlay. The respective collision cross-sections (CCS) of each ion, both with (blue font) and without (black font) QBP1 bound, are shown.

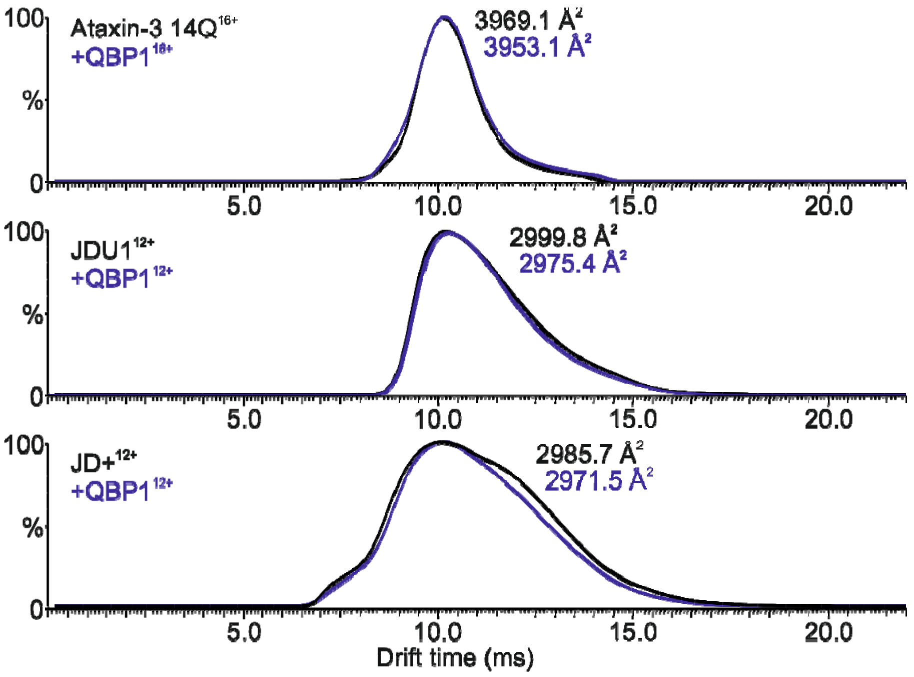

References

1. Blommel PG and Fox BG. A combined approach to improving large-scale production of tobacco etch virus protease. *Protein Expr. Purif.* 2007; 55: 53-68. doi: 10.1016/j.pep.2007.04.013

2. Compton LA and Johnson WC, Jr. Analysis of protein circular dichroism spectra for secondary structure using a simple matrix multiplication. *Anal. Biochem*. 1986; 155: 155-67. PMID: 3717552

3. Whitmore L and Wallace BA. DICHROWEB, an online server for protein secondary structure analyses from circular dichroism spectroscopic data. *Nucleic Acids Res.* 2004; 32: W668-73. doi: 10.1093/nar/gkh077

4. Song AX, Zhou CJ, Peng Y, et al. Structural transformation of the tandem ubiquitin-interacting motifs in ataxin-3 and their cooperative interactions with ubiquitin chains. *PloS one*. 2010; 5: e13202. doi: 10.1371/journal.pone.0013202

5. Scopes RK. Measurement of protein by spectrophotometry at 205 nm. *Anal. Biochem*. 1974; 59: 277-82. PMID:4407487
